# Religiosity is negatively associated with later-life intelligence, but not with age-related cognitive decline^☆^

**DOI:** 10.1016/j.intell.2014.04.005

**Published:** 2014-09

**Authors:** Stuart J. Ritchie, Alan J. Gow, Ian J. Deary

**Affiliations:** a Department of Psychology, The University of Edinburgh, United Kingdom; b Centre for Cognitive Ageing and Cognitive Epidemiology, The University of Edinburgh, United Kingdom; c Department of Psychology, School of Life Sciences, Heriot-Watt University, United Kingdom

**Keywords:** Religion, Intelligence, Cognitive decline, Latent growth curve

## Abstract

A well-replicated finding in the psychological literature is the negative correlation between religiosity and intelligence. However, several studies also conclude that one form of religiosity, church attendance, is protective against later-life cognitive decline. No effects of religious belief *per se* on cognitive decline have been found, potentially due to the restricted measures of belief used in previous studies. Here, we examined the associations between religiosity, intelligence, and cognitive change in a cohort of individuals (initial *n* = 550) with high-quality measures of religious belief taken at age 83 and multiple cognitive measures taken in childhood and at four waves between age 79 and 90. We found that religious belief, but not attendance, was negatively related to intelligence. The effect size was smaller than in previous studies of younger participants. Longitudinal analyses showed no effect of either religious belief or attendance on cognitive change either from childhood to old age, or across the ninth decade of life. We discuss differences between our cohort and those in previous studies – including in age and location – that may have led to our non-replication of the association between religious attendance and cognitive decline.

## Highlights

• Previous research has linked higher religiosity to lower intelligence.
• Paradoxically, religiosity has also been linked to healthier cognitive aging.
• We tested these relationships in a sample with cognitive tests across the ninth decade.
• Religious belief was negatively associated with intelligence in old age.
• Neither religious belief nor attendance was related to cognitive decline.

## 1 Introduction

Religiosity, measured by how often individuals attend religious ceremonies or by questionnaires assessing religious belief, has been consistently negatively associated with cognitive ability (Zuckerman, Silberman, & Hall, 2013). That is, individuals who are more religious tend to have lower intelligence, albeit by only a small degree. However, some studies indicate that, in later life, religiosity is protective against age-related cognitive decline (e.g. Van Ness & Kasl, 2003). In the present study, we investigate this apparent paradox in a sample of older individuals who completed detailed measures of religiosity at age 83 years, and for whom cognitive ability data were available from childhood and from multiple tests between ages 79 and 90.

Evidence for the negative relation of religiosity to cognitive ability comes from a variety of studies, recently meta-analyzed by Zuckerman et al. (2013). Over 85% of the 63 studies included in the analysis showed a negative correlation between the two measures, and the overall random-effects meta-analytic correlation between religiosity and intelligence was *r* = − .24. Zuckerman et al. (2013) discussed a number of possible explanations for this correlation, ranging from the lower propensity of high-IQ individuals to conform to religious dogma, to the possibility that religion acts to support attributes that higher intelligence may itself confer on other individuals, such as self-enhancement and self-control. Importantly for the present study, the majority (73%) of the studies in the meta-analysis examined religiosity and intelligence in university students or even younger samples, and only two studies – Blanchard-Fields, Hertzog, Stein, and Pak (2001), who studied a sample ranging from 23 to 86 years, and McCullough, Enders, Brion, and Jain (2005), who used longitudinal data following a sample aged 24–40 in 1940 across over 50 years – included some individuals who were in later life. To date, no studies have examined the correlation between religion and intelligence in individuals over 80 years of age. Since late life is a time at which individuals may engage in greater reflection on the past, with concomitant increases in religiosity (Hunsberger, 1985), it is of particular interest to test whether the association between intelligence and religiosity tends to be of a different size (or direction) in later life compared to earlier ages.

A smaller literature exists testing the relation of religion to later-life cognitive change. In one sample of 2812 older individuals aged 65 years and above (Van Ness & Kasl, 2003), higher religious attendance, but not stronger religious identity, was associated with lower rates of cognitive impairment 3 years later (but not by 6 years later) as measured on the Short Portable Mental Status Questionnaire (SPMSQ; Pfeiffer, 1975). A subsequent study (Hill, Burdette, Angel, & Angel, 2006) found a similar result in 3050 Mexican–Americans: those who attended church more regularly had shallower declines in cognitive function as measured by the Mini-Mental State Examination (MMSE; Folstein, Folstein, & McHugh, 1975) than those who were less frequent or non-attendees (see also Reyes-Ortiz et al., 2008, for a further analysis of the same dataset including an extra wave of cognitive testing, extending the study to 11 years, with the same conclusions). Yeager et al. (2006), in a sample of 4440 Taiwanese individuals, found effects of religious attendance (but, again, not belief) on cognitive decline measured by three cognitive tests, such that individuals with more regular attendance had better cognition after 4 years of follow-up. Finally, Corsentino, Collins, Sachs-Ericsson, and Blazer (2009) analyzed a sample of 2938 American women aged 65 and over across a three-year follow-up period, finding that religious attendance was associated with less cognitive decline, also measured using the SPMSQ.

The effect of religious attendance, but not belief, found in these studies is usually interpreted as indicating that social engagement, regardless of its type, is beneficial in cognitive aging (see e.g. Zunzunegui, Alvarado, Del Ser, & Otero, 2003); the beliefs *per se*, therefore, might be unimportant. For instance, Yeager et al. (2006) saw the relation of attendance to cognitive decline disappear in the presence of controls for social engagement. However, some studies have found effects of religious attendance even after controlling for social support (e.g. Corsentino et al., 2009), suggesting that specific aspects of religious activity are protective against cognitive decline. It is perhaps difficult, then, to reconcile these findings with the research that shows a relatively unambiguous negative correlation of religiosity with cognitive ability, discussed above, unless the intelligence–religion relationship is substantially different in old age.

The previous research on cognitive decline and religiosity has some limitations that may explain this apparent contradiction. First, all the studies, aside from that of Yeager et al. (2006), use cognitive function measures such as the MMSE and the SPMSQ that are designed to detect cognitive pathology. Such measures are useful for screening older individuals for dementia, but do not necessarily provide an accurate estimate of their general intelligence. In addition, these tests have reasonably pronounced ceiling effects, and thus tend to have poor sensitivity to milder cognitive decline, or cognitive decline in healthier or more highly educated samples (e.g. Pendlebury, Cuthbertson, Welch, Mehta, & Rothwell, 2010). Second, the studies that were able to assess religious belief in addition to attendance tend to have done so using very short, simple measures that may not have been sensitive enough to detect associations with change in cognition. It is still an open question, then, whether and how a more detailed measure of religious belief – tapping more dimensions of belief, and obtaining a better spread of scores than a one-item measure – would be associated with cognitive decline. Third, the follow-up periods of all the studies except Reyes-Ortiz et al. (2008) are less than 10 years. Fourth, all samples included a wide age range.

Here, we sought to overcome these limitations by analyzing a narrow-age cohort with multiple, detailed measures of religious belief and multiple, sensitive cognitive tests taken four times across an eleven-year period that covered the entire ninth decade of life. The cohort is also situated in the United Kingdom, a country with generally low religiosity (Norris & Inglehart, 2004), where no previous studies of religiosity and cognitive decline have been reported. It was therefore of interest to test whether the findings from previous studies held in a society in which relatively less importance is attached to religious attendance, and where older individuals may receive social support from other, non-religious social groups.

### 1.1 The present study

In line with the previous literature, we hypothesized that religiosity, measured in this study by religious belief and church attendance, would be significantly negatively correlated with intelligence. We then tested three hypotheses regarding religiosity's association with cognitive change. A rare aspect of our sample – the participants' completion of a test of cognitive ability in childhood as well as in old age – allowed us to test the hypothesis that religiosity would be associated with cognitive change across the life course (from age 11 to age 79). To our knowledge, no previous papers have tested this hypothesis. We also tested the hypothesis that longitudinal cognitive change would be associated with change in religiosity (in this sample, measured by religious attendance only). Finally, we tested the hypothesis that religiosity would be associated with cognitive change within the ninth decade of life. Based on the literature discussed above, the prediction was made that only religious attendance, and not religious belief, would associate with the slope of later-life cognitive change such that more regular attendance would be associated with shallower decline.

## 2 Method

### 2.1 Participants

Participants were members of the Lothian Birth Cohort 1921 (Deary et al., 2012, Deary et al., 2004), a longitudinal study of aging, especially cognition. Most of these individuals had completed a test of cognitive ability as part of the Scottish Mental Survey 1932 (Scottish Council for Research in Education, 1933), and were contacted for follow-up testing between 1999 and 2001, when they were aged an average of 79.1 years (SD = .6). At the first wave of testing in older age, 550 individuals (316 female) attended a clinical research facility at the Wellcome Trust Clinical Research Facility for assessments. The cohort has been followed up in three subsequent waves, in 2003–2005 (mean age = 83.4 years, SD = .5; *n* = 321), 2007–2008 (mean age = 86.6 years, SD = .4, *n* = 237) and 2011–2012 (mean age = 90.1 years, SD = .1; *n* = 129; Deary et al., 2012). In terms of religion, all participants were either Christian or non-religious.

Each participant was screened for possible dementia with the MMSE (Folstein et al., 1975); we excluded nine participants whose scores were below 24, a commonly-used cutoff for possible cognitive impairment, at age 79. Thus, the present study includes only those individuals with cognitive ability in the range often considered “healthy” at baseline.

### 2.2 Measures

#### 2.2.1 Cognitive ability

In 1932, the participants completed the Moray House Test (MHT) No. 12, a verbal reasoning-focused measure of cognitive ability that has been validated in childhood against the Stanford Revision of the Binet Test. For full details, see Deary et al. (2004).

At each of the four follow-up waves, cognitive ability was assessed using three tests: Raven's Standard Progressive Matrices (Raven, Court, & Raven, 1977), phonemic verbal fluency (Lezak, Howieson, & Loring, 2004), and logical memory from the Wechsler Memory Scale—Revised (Wechsler, 1987). For the analyses described below, a general, maximum-likelihood factor of cognitive ability (*g*) was extracted from these tests using Bartlett's method for scores. The percentage variance explained by this general factor was 31% at age 79, 35% at age 83, 38% at age 87, and 37% at age 90.

Participants were also administered the National Adult Reading Test (NART; Nelson & Willison, 1991). Note that the NART was not included as an indicator of the general factor since it is used to estimate cognitive ability prior to any later-life decline (McGurn et al., 2004); it was thus not appropriate for inclusion in our analyses of current cognitive functioning in older age, using tests that are sensitive to cognitive decline.

#### 2.2.2 Religiosity

At wave 1 (age 79), the participants completed an activity questionnaire, one item of which assessed how often they attended church, on the following four-point scale: 0, never; 1, rarely; 2, sometimes; 3, frequently. A similar measure using a different scale was administered to participants as part of a booklet-based survey mailed to participants (see Gow, Watson, Whiteman, & Deary, 2011, for details of this survey, which was returned by 444 participants). The question read: “how often do you usually attend religious ceremonies?” This was answered on a six-point scale (never/once a year or less/several times a year/once or more each month/once a week/more than once a week) that, for the purposes of the present study, was recoded to the same categories as the scale from wave 1: “never” remained “never”; “once a year or less” was recoded to “rarely”; “once or more each month” and “once a week” were recoded to “sometimes”; and “once a week” and “more than once a week” were recoded to “frequently.”

The age-83 booklet contained two detailed questionnaires assessing religious belief. The Religious Involvement Inventory (RII; Hilty & Morgan, 1985) assesses the influence of religion in an individual's life. Participants responded to each of 33 items on a 4-point scale. For the 10 items that were phrased in terms of frequencies (e.g. “how often do you read the Bible?”), this scale ranged from “never” to “regularly,” and for the 23 items that were statements of belief (e.g. “I know that God answers my prayers”) this scale ranged from “strongly agree” to “strongly disagree.” The RII is intended to include two subscales, “Orthodoxy” (19 items) and “Personal Faith” (14 items). However, the total scores from these two subscales correlated very highly (*r*(340) = .86, *p* < .001), and the Cronbach's α across all the items was .98 (see also Gow et al., 2011, for evidence that the two factors are not distinct). We thus chose to extract, using the same procedure as that for the cognitive tests described above, a single, unrotated factor (“general religious involvement”) from the inventory. This factor explained 60.9% of the variance across the items.

The Spiritual Wellbeing Scale (SWBS; Paloutzian & Ellison, 1982) measures two factors, “religious wellbeing” and “existential wellbeing,” each with 10 items. For the present study, we used only the “religious wellbeing” items, since the “existential” items contain no questions regarding religiosity. Two example items from the “religious wellbeing” scale are “I have a personally meaningful relationship with God” and “I believe that God loves me and cares about me.” All items were answered on a 6-point scale, from “strongly disagree” to “strongly agree,” and the Cronbach's α of the scale was .95. A single factor extracted from the “religious wellbeing” items explained 65.00% of their variance, and this was used in the calculations described below.

#### 2.2.3 Covariates

Analyses included covariates of education and socioeconomic status (SES). At interview at wave 1, participants indicated their number of years of formal, full-time education. They were asked about highest status job they had before retiring, which was placed on a social class scale from class V (unskilled) to I (professional), according to the 1951 Classification of Occupations from the General Register Office Census (General Register Office, 1956).

### 2.3 Statistical analysis

We first tested the bivariate correlations among the cognitive, religiosity, and demographic variables. Power analysis indicated that, with our valid sample size of 339 at age 83, we had 99% power to detect effect sizes similar to that found in the Zuckerman et al. (2013) meta-analysis (*r* = (−).24), and 80% power to detect effects of *r* = (−).15. Where measures only from age 79 were used, our sample sizes were somewhat larger.

Before their inclusion in any of the models, the cognitive test scores were controlled for age (in days) at the time of testing. We then used multiple linear regression to control for the influences of the covariates, and tested the association of the religiosity variables with the measures of cognitive ability at each age. We included sex as a covariate since reasonably consistent sex differences in religiosity have been found in previous studies (with females tending to be more religious; Francis & Penny, 2014). We also controlled for education, since education has been found to correlate with both religiosity and intelligence in previous studies (e.g. Lewis, Ritchie, & Bates, 2011), and socioeconomic status, since participants of different social classes may have had differences in their involvement in religious activities for non-cognitive reasons.

Finally, to test associations between religious measures and cognitive change within later life, we used latent growth curve models, fit by full-information maximum likelihood estimation, using the “OpenMx” package (Boker et al., 2011) for R with Bates's (2013) additional “umx” functions. The paths of interest in the model were those between the covariate of religiosity (measured in the three ways described above: religious involvement, religious wellbeing, and attendance) and the latent intercept and slope of general cognitive ability. To set the metric of each general intelligence factor, one loading at each wave was fixed at 1. We assumed factorial invariance, setting the intercepts of each cognitive test, their loadings on their general factor, and the residual factor variances to be the same across waves (the findings of interest described below were not substantively different if these constraints were not applied). The growth curve models also included the covariates described above.

## 3 Results

Table 1 shows descriptive statistics for each of the religiosity variables and general cognitive ability at each testing wave, and Table 2 shows their bivariate correlations after correction for age. The religiosity variables were highly correlated with one another (all above *r* = .6). The correlations between religious belief and intelligence measured at all four waves were negative; however, only the correlation with intelligence at age 79 (4 years before the religiosity questionnaires were completed; *r* = − .15 for involvement and *r* = − .14 for wellbeing) was significant. None of the measures of intelligence were correlated with religious attendance. There was also no relation of the two measures of pre-existing intelligence (age 11 MHT and NART) to any of the religiosity variables; only later-life intelligence was correlated with religious belief in our sample. However, educational duration was significantly positively related to age 83 religious attendance (*r* = .12; but not age 79 attendance: r = .03), while socioeconomic status was negatively related to religious wellbeing (*r* = − .12).

**Table 1:** Descriptive statistics and sample sizes for each of the cognitive, religiosity, and control variables. Maximum scores are provided in parentheses after each variable name.

Measure (max. score)	*n* Age 79/83/87/90	Mean (SD) age 79	Mean (SD) age 83	Mean (SD) age 87	Mean (SD) age 90
Religious Involvement Inventory (132)	–/360/–/–	–	73.94 (22.70)	–	–
SWBS — Religious Wellbeing (60)	–/338/–/–	–	36.48 (12.32)	–	–
Religious attendance (4)	466/360/–/–	1.90 (1.19)	1.84 (1.23)	–	–
Moray House Test age 11 (76)	483	–	46.63 (11.92)	–	–
SES (5)	537/–/–/–	2.23 (.87)	–	–	–
Education	537/–/–/–	10.93 (2.47)	–	–	–
NART (50)	537/–/–/–	34.33 (8.16)	–	–	–
Raven's Matrices (60)	532/312/200/116	31.38 (8.65)	29.85 (9.13)	27.89 (9.20)	26.39 (8.58)
Verbal fluency	536/315/205/124	40.19 (12.25)	39.91 (12.77)	40.04 (12.31)	39.59 (13.32)
Logical memory (50)	539/288/205/126	31.94 (12.66)	32.80 (14.75)	33.02 (14.60)	33.27 (16.69)
General cognitive ability (*g*)	529/286/200/116	.14 (1.18)	− .02 (1.33)	− .18 (1.33)	− .29 (1.30)

Note: General cognitive ability (*g*) is a latent variable calculated from the scores on Raven's Matrices, verbal fluency, and logical memory at each age.

**Table 2:** Correlation matrix for religiosity, cognitive, and control variables.

	1.	2.	3.	4.	5.	6.	7.	8.	9.	10.	11.	12.
1. Religious involvement	–											
2. Religious wellbeing	**.90**	–										
3. Religious attendance age 79	**.64**	**.58**	–									
4. Religious attendance age 83	**.70**	**.62**	**.84**	–								
5. MHT age 11	− .02	− .05	.00	.07	–							
6. NART age 79	− .02	− .05	− .01	.07	**.66**	–						
7. *g* age 79	− .15⁎⁎	− .14⁎	− .01	.01	**.48**	**.53**	–					
8. *g* age 83	− .09	− .09	− .04	.00	**.53**	**.51**	**.84**	–				
9. *g* age 87	− .07	− .08	.02	.03	**.43**	**.44**	**.72**	**.86**	–			
10. *g* age 90	− .19	− .16	− .01	− .06	**.50**	**.42**	**.63**	**.79**	**.76**	–		
11. Education	− .03	− .06	.03	.12⁎	**.43**	**.53**	**.37**	**.37**	**.36**	**.33**	–	
12. SES	− .09	− .12⁎	.05	.07	**.40**	**.49**	**.30**	**.29**	.23⁎⁎	.26⁎⁎	**.48**	–
13. Sex	**.28**	**.23**	**.21**	.16⁎⁎	.02	− .01	− .13⁎⁎	− .07	.04	.09	− .12⁎⁎	− .12⁎⁎

Note: Bold coefficients = *p* < .001. All cognitive tests corrected for age in days at testing. Religious involvement = Religious Involvement Inventory general factor; religious wellbeing = Spiritual Wellbeing Scale–religious wellbeing score; MHT = Moray House Test; NART = National Adult Reading Test; *g* = general cognitive ability, SES = socioeconomic status.

⁎ *p* < .05.

⁎⁎ *p* < .01.

### 3.1 Regression analyses

Table 3 shows the multiple linear regression results for three models with each religiosity variable as the outcome and intelligence at age 79 (to maximize the valid sample size and therefore the power to detect a result) plus the covariates as predictors. Only in the first analysis, with the RII factor as the outcome, did intelligence have a significant (negative) association. That is, the RII factor was related to the general cognitive ability factor (*β* = − .14), but there were no significant associations for religious wellbeing or attendance (note that, for each analysis below, attendance at age 79 was the variable used unless otherwise stated; no substantial differences were found if this variable was replaced with attendance at age 83). For intelligence measured at ages 83 and 87, the RII factor had no significant relationship to intelligence. Overall, then, the associations that were significant in the correlation matrix remained so after correction for sex, education, and SES, and this adjustment did not cause any of the non-significant correlations become significant.

**Table 3:** Multiple linear regression results for three regressions predicting each of the three religiosity variables.

Outcome (valid *n*)	Predictor	*β*	SE	*t*-Value	*p*-Value
Religious involvement (339)	Intercept	− .28	.08	− 3.33	< .001
	Sex — female	.53	.11	4.98	< .001
	SES	− .07	.06	− 1.06	.29
	Education	.08	.06	1.28	.20
	General intelligence	− .14	.06	− 2.36	.02
Religious wellbeing (336)	Intercept	− .22	.08	− 2.61	.01
	Sex — female	.43	.11	3.94	< .001
	SES	− .10	.06	− 1.49	.14
	Education	.05	.06	.84	.40
	General intelligence	− .12	.06	− 1.86	.06
Attendance (455)	Intercept	− .25	.07	− 3.54	< .001
	Sex — female	.44	.09	4.68	< .001
	SES	.07	.05	1.32	.19
	Education	.04	.05	.70	.49
	General intelligence	− .02	.05	− .42	.67

Note: All cognitive variables adjusted for age in days before inclusion in the analyses, and measured at age 79 (to maximize valid sample size). Religious involvement = Religious Involvement Inventory general factor; religious wellbeing = Spiritual Wellbeing Scale–religious wellbeing; SES = socioeconomic status.

We then tested our second hypothesis, regarding the association between religiosity and cognitive change across the life course (from age 11 to age 83). We first regressed age 83 general intelligence on age 11 Moray House Test score and saved the residuals as an index of lifetime cognitive change. We then used this index as the outcome in a regression model including the control variables and all three religiosity variables. The results of this model are shown in Table 4; there were no significant effects of any of the religiosity variables on cognitive change across the life course, and this remained the case in three subsequent models with each of the religiosity variables entered alone. Thus, we found no significant effect of religiosity – of any sort – on cognitive change from childhood to old age.

**Table 4:** Multiple linear regression results for a model (valid *n* = 226) predicting lifetime cognitive change (between age 11 and age 83).

Predictor	*β*	SE	*t*-Value	*p*-Value
Intercept	.09	.10	.96	.34
Sex — female	.02	.13	.12	.90
SES	.02	.08	.26	.79
Education	.17	.07	2.32	.02
Religious involvement	.03	.16	.18	.85
Religious wellbeing	− .04	.15	− .27	.79
Religious attendance	.07	.09	− .87	.39

Note: All cognitive variables adjusted for age in days before inclusion in the analyses. Religious involvement and wellbeing measured at age 83; religious attendance measured at age 79. Religious involvement = Religious Involvement Inventory general factor; religious wellbeing = Spiritual Wellbeing Scale–religious wellbeing; SES = socioeconomic status.

Our third hypothesis was tested by obtaining an index of change in religious attendance and general cognitive ability between ages 79 and 83 (again by regressing the later score on the earlier score and saving the residuals) and using attendance change to predict cognitive change in a regression model controlling for the covariates (sex, SES, and education). Paired *t*-tests showed that there was significant decline in both age-corrected general cognitive ability (*t*(284) = 4.94, *p* < .001, *d* = .05) and religious attendance (*t*(344) = 3.12, *p* < .001, *d* = .08) between ages 79 and 83. The regression results, shown in Table 5, showed no significant effects of any of the variables; most importantly, change in religious attendance was not associated with change in general cognitive ability.

**Table 5:** Multiple linear regression results for a model (valid *n* = 247) predicting cognitive change between ages 79 and 83.

Predictor	*β*	SE	*t*-Value	*p*-Value
Intercept	− .07	.09	− .75	.46
Sex — female	.17	.12	1.34	.18
SES	− .03	.07	− .47	.64
Education	.06	.07	.91	.36
Religious attendance change	− .02	.06	− .39	.70

Note: All cognitive variables adjusted for age in days before inclusion in the analyses. SES = socioeconomic status.

### 3.2 Latent growth curve analyses

**Fig. 1:**
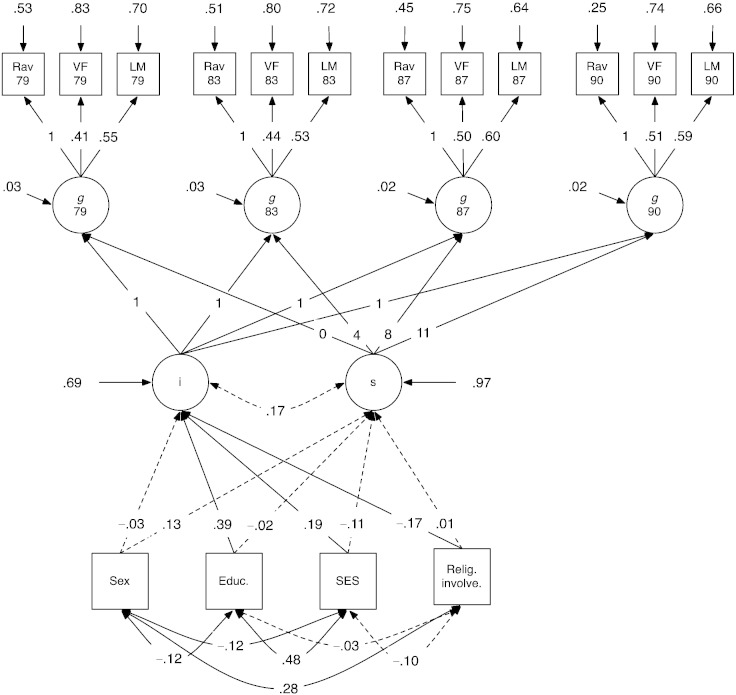
Latent growth curve model of cognitive change, with religious involvement. Values are standardized path coefficients. Dotted lines indicate non-significant paths (could be dropped without significant decrement in model fit). Residual longitudinal correlations between cognitive tests not shown for reasons of space; see Table A1. Note: i = latent intercept; s = latent slope; *g* = general factor of cognitive ability; Rav = Raven's Standard Progressive Matrices; VF = verbal fluency; LM = logical memory; Relig. Involve. = religious involvement (general factor); Educ. = years of education; SES = socioeconomic status. For sex, 0 = male and 1 = female.

Our final hypothesis, in line with several previous studies, stated that higher religiosity would protect against cognitive decline in old age. We entered the cognitive variables from the four later-life waves into three latent growth curve models (one for each of the religiosity variables), allowing the religiosity variable and the covariates (sex, socioeconomic status, and years of education) to have paths to the intercept and slope of cognitive ability, as well as residual correlations with one another. The model for the RII factor variable is shown in Fig. 1, and the values for the longitudinal correlations between subtests are shown in Table A1 in the Appendix A. All three models fit well to the data (RII: *χ*^2^(5390) = 121.19, root mean square error of approximation = .03, comparative fit index = .99, Tucker–Lewis index = .98; religious wellbeing: *χ*^2^(5386) = 120.92, RMSEA = .03, CFI = .99, TLI = .98; attendance: *χ*^2^(5408) = 116.24, RMSEA = .02, CFI = .99, TLI = .98).

As can be seen in Fig. 1, whereas religious involvement was associated with the intercept of cognitive ability, it was not associated with the slope. Therefore, religious involvement was associated with average level of cognitive ability, but was not protective against cognitive decline. Confidence intervals around each of the theoretically-relevant paths in the three models are shown in Table 6. For religious wellbeing, a smaller, non-significant association with the intercept of cognitive ability was found (coefficient = − .13, 95% CI [− .27, .004]), and there was a yet smaller link with religious attendance (coefficient = − .04, [− .18, .09]). None of the religiosity measures were significantly associated with the slope of cognitive change. Overall, then, we could find no evidence that religiosity affected cognitive decline within old age. Similarly, none of the control variables had significant relations to the slope of cognitive decline, but there were substantial associations between educational duration and SES and the intercept in each model (coefficients = .39 [.26, .51] and .19 [.08, .30] respectively in the model with religious involvement; effects were similar in the remaining two models).

**Table 6:** Standardized path coefficients and standard errors, with 95% confidence intervals, for the covariate relations to cognitive intercept and slope, for the three models (“Religious involvement” model shown in Fig. 1). Statistically significant coefficients (*p* < .05) are in bold.

Model	Path from	Path to	Coefficient	SE	95% CI
Religious involvement	Sex	Intercept	− .03	.05	[− .13, .07]
	Sex	Slope	.13	.09	[− .05, .30]
	Education	Intercept	**.39**	.06	[.28, .51]
	Education	Slope	.02	.09	[− .16, .19]
	SES	Intercept	**.19**	.06	[.08, .30]
	SES	Slope	− .11	.10	[− .31, .09]
	Religiosity	Intercept	**− .17**	.07	[− .30, − .03]
	Religiosity	Slope	.01	.10	[− .20, .18]
Religious wellbeing	Sex	Intercept	− .04	.05	[− .15, .06]
	Sex	Slope	.12	.09	[− .05, .30]
	Education	Intercept	**.39**	.06	[.27, .51]
	Education	Slope	− .01	.09	[− .16, .19]
	SES	Intercept	**.19**	.06	[.08, .31]
	SES	Slope	− .11	.10	[− .31, .09]
	Religiosity	Intercept	− .13	.07	[− .27, .004]
	Religiosity	Slope	.02	.09	[− .16, .20]
Religious attendance	Sex	Intercept	− .07	.05	[− .17, .04]
	Sex	Slope	.15	.09	[− .02, .32]
	Education	Intercept	**.39**	.07	[.27, .51]
	Education	Slope	.02	.09	[− .15, .20]
	SES	Intercept	**.21**	.06	[.09, .33]
	SES	Slope	− .11	.10	[− .30, .09]
	Religiosity	Intercept	− .04	.07	[− .18, .09]
	Religiosity	Slope	− .09	.09	[− .27, .09]

Note: Religious involvement and wellbeing measured at age 83; religious attendance measured at age 79.

## 4 Discussion

In a sample of adults tested in childhood and across the ninth decade of life, we examined the associations of religiosity – measured with two scales of religious belief and an indicator of attendance at religious ceremonies – with cognitive ability, cognitive change across the lifespan, and cognitive change within later life. In our regression analyses, we replicated the direction of the effect found in previous studies: religiosity–intelligence associations were always negative. However, in general, we found smaller relationships than indicated by a meta-analysis of this topic (Zuckerman et al., 2013). Whereas religiosity was significantly negatively associated with intelligence measured 4 years earlier, the association with a concurrent intelligence measure, while negative, did not reach significance (this may have been due to the lower valid sample size, and thus lower power, for this correlation). There was no evidence of religiosity's association with cognitive change either from age 11 to age 79, from age 79 to age 83, or from age 79 to age 90. We did not, therefore, find evidence for the somewhat paradoxical phenomenon described above, where religiosity is associated with lower cognitive ability but religious attendance protects against cognitive decline in older age.

Our estimates of the covariate-adjusted relation of general intelligence to religious belief (*β =* − .14, *p* = .02 for the general factor from the Religious Involvement Inventory, and *β =* − .12, *p* = .06 for the religious wellbeing factor from the Spiritual Wellbeing Scale) were on the lower end of the effect sizes taken from the meta-analysis of religion and intelligence by Zuckerman et al. (2013), which produced an overall effect size of *r* = − .24. Indeed, the correlations between intelligence tests and religiosity were generally small and non-significant (Table 2), though the association between religious involvement and the overall level of cognitive ability across all waves in our growth curve model was significant; this may have been due to the enhanced power afforded by the maximum-likelihood modeling approach. In any case, the effect sizes were modest; as was suggested above, these smaller effects may be due to those individuals lower in religion at earlier ages becoming more religious as they age, restricting the range in religiosity and thus attenuating the correlation with intelligence.

A similar explanation may underlie our finding of a small, non-significant association between religious attendance and intelligence (*β =* − .02, *p* = .67); due to its social aspects, religious attendance may at later ages become more habitual for a larger percentage of the population, causing it to lose its value as an indicator of intelligence. Alternatively, these findings may be cohort effects: for older cohorts, born at times of higher societal religiosity (see Norris & Inglehart, 2004, p. 77, Fig. 3.4), attendance at religious ceremonies may be a weaker signal of cognitive ability. A longitudinal study testing both religiosity and cognitive ability multiple times from midlife into old age would be useful to test between these possibilities.

It should be noted that our results do not shed light on the reason for the negative correlation of religious belief and intelligence, now known to extend into late life. Future studies should attempt to tease apart the potential reasons for the correlation discussed by Zuckerman et al. (2013), some of which are outlined above. It may be particularly interesting to assess whether there are different explanations for the correlation at different stages in the life course, and whether, for instance, younger individuals with lower intelligence are more likely to be religious due to conformism whereas older low-intelligence individuals are more likely to be religious due to religion's propensity to resolve uncertainty.

Consistent with the previous literature on religion and cognitive decline, we found no effects of religious belief on the slope of change in cognitive ability in old age. This was despite our inclusion of two measures of religious belief that were more comprehensive than in any study of this question to date. Including our study, then, no research to date has found any evidence that individuals who hold stronger religious beliefs will be protected against cognitive decline. However, our study was not consistent with the finding of all previous studies in this area (e.g. Corsentino et al., 2009, Hill et al., 2006, Van Ness and Kasl, 2003), that attendance at religious ceremonies was associated with healthier cognitive aging. We found no effect of religious ceremony attendance on cognitive change either across the lifespan, or during later life. It should be noted that, to our knowledge, this is the first study to test the religion–cognitive decline association decline in a UK population. It is plausible that our conflicting results are due to the generally lower levels of importance placed on religion in the UK compared to, for example, the US (see Norris & Inglehart, 2004, Ch. 4 for international comparisons), where most previous studies have been carried out (though see Yeager et al., 2006). This highlights an important nuance for this area of study: in samples from more secularized countries, such as those in Western Europe, religiosity may have smaller health effects than in countries where religion remains an important part of life.

Although religious belief and attendance had either negative or non-significant relations to the cognitive variables, religious attendance was significantly positively correlated with educational duration (*r* = .12, Table 1). This is perhaps surprising, since each of the cognitive variables themselves had a moderate-to-strong positive correlation with education. We would speculate that, in the UK, older individuals with higher education may gravitate toward the social aspects of religion, regardless of their intelligence. This is partly supported by the positive (though admittedly non-significant) correlation of SES with religious attendance; conversely, SES was negatively related to the measures of religious belief. Further studies of the correlates of the various dimensions of religiosity in later life should be performed to address these differential effects in the social and cognitive domains.

As noted above, the strengths of this study were in our detailed measures of religious belief and in the length, frequency, detail, and age of follow-up testing. We were able to examine the associations of religiosity with cognitive decline across the ninth decade of life in a narrow-age cohort, greatly reducing any problems of age heterogeneity that may have influenced the results of previous studies. In addition, we were not reliant on basic, pathology-focused tests of cognitive function such as the MMSE, but estimated a general factor of ability from multiple tests at each age.

The study has limitations. First, the sample size is modest. Whereas we were adequately powered to reliably detect religion–intelligence correlations of the size found in the Zuckerman et al. (2013) meta-analysis, it is possible that we were unable to detect smaller effects of religiosity on cognitive decline in our growth curve analysis. The growth curve would also have benefitted from a simultaneous model of latent change in religious belief from age 79 to age 90, allowing an estimation of the correlation of changes in religiosity with those in cognition; longitudinal, latent measurements of religiosity were not available in this sample. Our analysis of change in religious attendance between 79 and 83 was restricted by the attendance measures both comprising one item; a range of items would have allowed a more complex, latent-difference score analysis (e.g. McArdle, 2009). This was also a limitation for our analysis of lifetime cognitive change: no measures of religiosity were available from earlier than age 79, and no measures of religious belief from earlier than 83, meaning that the religiosity measurements were taken after the lifetime cognitive change had occurred. Since, as noted above, religiosity changes throughout the lifespan, it is not safe to assume that religiosity levels in older age reflect those earlier in life. A study with multiple religiosity and cognitive measures taken across the lifespan would be able to provide more accurate results on this question.

Further, our cohort had a restricted range of intelligence, tending to have somewhat higher and less varied intelligence scores than the general population (Deary, Whalley, Lemmon, Crawford, & Starr, 2000). If religiosity is particularly protective against cognitive decline in those toward the lower end of the intelligence distribution, we may have missed its effects. Finally, our religious belief measures were high quality, but our measure of religious attendance was restricted to a single item. More detailed measures, perhaps taking into account the depth of and reasons for an individual's religious attendance, would perhaps produce different results.

## 5 Conclusion

The present study examined the associations between religiosity, intelligence, and cognitive change across the lifespan and in later life. It replicated the negative intelligence–religiosity correlation found in previous studies (which almost always included younger participants), though with smaller effect sizes than have been found previously. Tentatively, we suggest that this difference may reflect the higher average religiosity level in older cohorts. However, the religiosity levels in the cohort still had no effect on change in cognition across the lifespan or within later life: we failed to replicate the finding, discovered in all previous studies, that more frequent attendance at religious ceremonies is associated with healthier cognitive aging. We recommend that future studies of religiosity and later-life cognitive decline take into account the possibility that, in countries with less overall societal influence of religion, spiritual and religious activity may exert a reduced influence on cognitive aging.

## Figures and Tables

**Table A1:** Standardized path coefficients for residual longitudinal correlations between cognitive tests (not shown in Fig. 1). These values were near-identical across all growth curve models.

Variable 1 (age)	Variable 2 (age)	Coefficient	SE
Rav (79)	Rav (83)	.34	.06
Rav (79)	Rav (87)	.32	.06
Rav (79)	Rav (90)	.26	.06
Rav (83)	Rav (87)	.31	.07
Rav (83)	Rav (90)	.23	.07
Rav (87)	Rav (90)	.18	.07
VF (79)	VF (83)	.60	.05
VF (79)	VF (87)	.59	.05
VF (79)	VF (90)	.57	.06
VF (83)	VF (87)	.61	.06
VF (83)	VF (90)	.58	.06
VF (87)	VF (90)	.57	.07
LM (79)	LM (83)	.49	.05
LM (79)	LM (87)	.39	.05
LM (79)	LM (90)	.33	.06
LM (83)	LM (87)	.48	.06
LM (83)	LM (90)	.38	.07
LM (87)	LM (90)	.45	.07

Note: Rav = Raven's Standard Progressive Matrices score; VF = verbal fluency; LM = logical memory.

## References

[bb0005] Bates T.C. (2013). umx: A help package for structural equation modeling in OpenMx. v. 0.6. http://github.com/tbates/umx/.

[bb0015] Blanchard-Fields F., Hertzog C., Stein R., Pak R. (2001). Beyond a stereotyped view of older adults' traditional family values. Psychology and Aging.

[bb0010] Boker S., Neale M., Maes H., Wilde M., Spiegel M., Brick T. (2011). OpenMx: An open source extended structural equation modeling framework. Psychometrika.

[bb0020] Corsentino E.A., Collins N., Sachs-Ericsson N., Blazer D.G. (2009). Religious attendance reduces cognitive decline among older women with high levels of depressive symptoms. The Journals of Gerontology Series A: Biological Sciences and Medical Sciences.

[bb0030] Deary I.J., Gow A.J., Pattie A., Starr J.M. (2012). Cohort profile: the Lothian Birth Cohorts of 1921 and 1936. International Journal of Epidemiology.

[bb0035] Deary I.J., Whalley L.J., Lemmon H., Crawford J.R., Starr J.M. (2000). The stability of individual differences in mental ability from childhood to old age: Follow-up of the 1932 Scottish Mental Survey. Intelligence.

[bb0040] Deary I.J., Whiteman M.C., Starr J.M., Whalley L.J., Fox H.C. (2004). The impact of childhood intelligence on later life: Following up the Scottish mental surveys of 1932 and 1947. Journal of Personality and Social Psychology.

[bb0045] Folstein M.F., Folstein S.E., McHugh P.R. (1975). “Mini-Mental State”: A practical method for grading the cognitive state of patients for the clinician. Journal of Psychiatric Research.

[bb0050] Francis L.J., Penny G., Saroglou V. (2014). Religion, personality, and social behavior.

[bb9000] General Register Office (1956).

[bb0055] Gow A.J., Watson R., Whiteman M., Deary I.J. (2011). A stairway to heaven? Structure of the religious involvement inventory and spiritual well-being scale. Journal of Religion and Health.

[bb0060] Hill T.D., Burdette A.M., Angel J.L., Angel R.J. (2006). Religious attendance and cognitive functioning among older Mexican Americans. The Journals of Gerontology Series B: Psychological Sciences and Social Sciences.

[bb0065] Hilty D.M., Morgan R.L. (1985). Construct validation for the religious involvement inventory: Replication. Journal for the Scientific Study of Religion.

[bb0070] Hunsberger B. (1985). Religion, age, life satisfaction, and perceived sources of religiousness: A study of older persons. Journal of Gerontology.

[bb0075] Lewis G.J., Ritchie S.J., Bates T.C. (2011). The relationship between intelligence and multiple domains of religious belief: Evidence from a large adult US sample. Intelligence.

[bb0080] Lezak M.D., Howieson D.B., Loring D.W. (2004).

[bb0085] McArdle J.J. (2009). Latent variable modeling of differences and changes with longitudinal data. Annual Review of Psychology.

[bb0090] McCullough M.E., Enders C.K., Brion S.L., Jain A.R. (2005). The varieties of religious development in adulthood: A longitudinal investigation of religion and rational choice. Journal of Personality and Social Psychology.

[bb0095] McGurn B., Starr J.M., Topfer J.A., Pattie A., Whiteman M.C., Lemmon H.A. (2004). Pronunciation of irregular words is preserved in dementia, validating premorbid IQ estimation. Neurology.

[bb9005] Nelson H.E., Willison J. (1991).

[bb0100] Norris P., Inglehart R. (2004).

[bb0105] Paloutzian R.F., Ellison C.W., Peplau L.A., Perlman D. (1982). Loneliness: A sourcebook of current theory, research and therapy.

[bb0110] Pendlebury S.T., Cuthbertson F.C., Welch S.J., Mehta Z., Rothwell P.M. (2010). Underestimation of cognitive impairment by Mini-Mental State Examination versus the Montreal Cognitive Assessment in patients with transient ischemic attack and stroke: A population-based study. Stroke.

[bb0115] Pfeiffer E. (1975). A short portable mental status questionnaire for the assessment of organic brain deficit in elderly patients. Journal of the American Geriatrics Society.

[bb0120] Raven J.C., Court J.H., Raven J. (1977).

[bb0125] Reyes-Ortiz C.A., Berges I.M., Raji M.A., Koenig H.G., Kuo Y.F., Markides K.S. (2008). Church attendance mediates the association between depressive symptoms and cognitive functioning among older Mexican Americans. The Journals of Gerontology Series A: Biological Sciences and Medical Sciences.

[bb0130] Scottish Council for Research in Education (1933).

[bb0135] Van Ness P.H., Kasl S.V. (2003). Religion and cognitive dysfunction in an elderly cohort. The Journals of Gerontology Series B: Psychological Sciences and Social Sciences.

[bb0140] Wechsler D. (1987).

[bb0145] Yeager D.M., Glei D.A., Au M., Lin H.S., Sloan R.P., Weinstein M. (2006). Religious involvement and health outcomes among older persons in Taiwan. Social Science & Medicine.

[bb0150] Zuckerman M., Silberman J., Hall J.A. (2013). The relation between intelligence and religiosity: A meta-analysis and some proposed explanations. Personality and Social Psychology Review.

[bb0155] Zunzunegui M.V., Alvarado B.E., Del Ser T., Otero A. (2003). Social networks, social integration, and social engagement determine cognitive decline in community-dwelling Spanish older adults. The Journals of Gerontology Series B: Psychological Sciences and Social Sciences.

